# M100 ExaData: a data collection campaign on the CINECA’s Marconi100 Tier-0 supercomputer

**DOI:** 10.1038/s41597-023-02174-3

**Published:** 2023-05-18

**Authors:** Andrea Borghesi, Carmine Di Santi, Martin Molan, Mohsen Seyedkazemi Ardebili, Alessio Mauri, Massimiliano Guarrasi, Daniela Galetti, Mirko Cestari, Francesco Barchi, Luca Benini, Francesco Beneventi, Andrea Bartolini

**Affiliations:** 1 Alma Mater Research Center for Human-Centered Artificial Intelligence, Bologna, 40121 Italy; 2 University of Bologna, DISI, Viale Risorgimento 2, Bologna, 40123 Italy; 3 CINECA, 40033 Casalecchio di Reno (BO), Casalecchio di Reno, Italy; 4 Integrated Systems Laboratory at ETH Zurich, Zurich, Switzerland

**Keywords:** Computer science, Information technology

## Abstract

Supercomputers are the most powerful computing machines available to society. They play a central role in economic, industrial, and societal development. While they are used by scientists, engineers, decision-makers, and data-analyst to computationally solve complex problems, supercomputers and their hosting datacenters are themselves complex power-hungry systems. Improving their efficiency, availability, and resiliency is vital and the subject of many research and engineering efforts. Still, a major roadblock hinders researchers: dearth of reliable data describing the behavior of production supercomputers. In this paper, we present the result of a ten-year-long project to design a monitoring framework (EXAMON) deployed at the Italian supercomputers at CINECA datacenter. We disclose the first holistic dataset of a tier-0 Top10 supercomputer. It includes the management, workload, facility, and infrastructure data of the Marconi100 supercomputer for two and half years of operation. The dataset (published via Zenodo) is the largest ever made public, with a size of 49.9TB before compression. We also provide open-source software modules to simplify access to the data and provide direct usage examples.

## Background & Summary

High-Performance Computing (HPC) systems are complex machines composed of a huge number of heterogeneous components, such as computing nodes with thousands of parts, cooling infrastructures, network connectors, and software elements. Over the years, the complexity of supercomputers and datacenters is increased following the growing demands of performance–and it is expected that it will keep on growing. This complexity brings along a series of big challenges for facility owners, system administrators, and practitioners. As an example of such challenges, we recall predictive maintenance (e.g., detection and prevention of faults), energy and power consumption, and workload management. The magnitude of the problem motivates the adoption of automated approaches to cope with these issues, especially data-driven solutions. Efficient and reliable HPC systems (and datacenters in general) are very important for society at large, as their usage has been steadily increasing in recent years^1^ making it central in a wide variety of fields, from drug design^2^ to precise weather forecast^3^ and crowd flow simulation^4^, especially in conjunction with Artificial Intelligence (AI)^5,6^. Their relevance will only become greater in future years, as demonstrated, for instance, by the successful impact supercomputers had in helping to quickly tackle the COVID-19 pandemic^7,8^ or make progress in fusion energy development^9^, and climate change^10^.

This socio-economical significance strongly motivates the research of novel methodologies to improve supercomputers’ maintenance, efficiency, and productivity. For this reason, researchers and practitioners need to precisely understand the behavior of supercomputers, with the goal of building data-driven predictive models, optimized policies, and digital twins. Public data in this field is very scarce and mostly kept on premises–whereas there is abundant availability of sensors at all-level of software, hardware and infrastructure, these are either (i) not stored and accessed on-demand for inspection, (ii) collected for instantaneous visualization but not preserved, or (iii) stored in different databases and logs, by different organizations (facility, user-support, system administrators) for post-mortem analysis^11^; hence, we decided to holistically measure and publish a large, exhaustive, and consistent dataset collected for two and half years on a Tier-0 system (the largest to the best of our knowledge). The dataset is the result of a ten-year collaboration project with the Italian supercomputing center, CINECA–hosted at Bologna, Italy (https://www.cineca.it/en)–on holistic monitoring of supercomputer infrastructure to provide innovation in green, autonomous, efficient datacenters and supercomputing centers. Throughout the years, we observed, studied, designed, and deployed a holistic, fine-grained, and comprehensive data collection infrastructure, which we have dubbed ExaMon^11,12^.

We now make available the entire data collected from the Tier-0 supercomputer hosted at CINECA (Marconi100–https://www.hpc.cineca.it/hardware/marconi100). The data covers the entirety of the system, including the computing nodes internal information such as core loads, temperatures, frequencies, memory write/read operations, CPU power consumption, fan speed, GPU usage details, etc.; (we cover *all* 980+ computing nodes). We consider as well the system-wide information, including the liquid cooling infrastructure, the air conditioning system, the power supply units, workload manager statistics, and job-related information, system status alerts, and weather forecast. The data collection period spans the last two and a half years. It comprises hundreds of metrics measured on each computing node, in addition to hundreds of other metrics gathered from sensors monitored along all system components, totaling up to 49.9 TB of (uncompressed) storage space. To the best of our knowledge, this is the largest public dataset of this kind in the supercomputing and datacenter community. Along with the dataset we provide a metadata repository consisting of a detailed description of the data, working scripts, and two use cases: room-level thermal hazard prediction and node-level unsupervised anomaly detection.

The main goal behind the publication of this dataset is to foster the research and development of solutions for data-driven sustainable high-performance computing and datacenters development–where sustainability include all aspects of operational management to counteract the increasing system complexity, in efficiency to counteract the increasing cost, and in carbon footprint to mitigate the growing cooling and operational energy demand.

## Methods

In this section we describe: I) how the data has been collected from the target supercomputer and briefly describe the monitoring infrastructure (Sec. “Data collection framework: ExaMon”), II) how the data has been extracted and the dataset prepared (Sec. “Dataset preparation”), III) how the data is then processed to remove redundant and/or sensitive information (Sec. “Data processing”), and IV) finally we provide some details on the fine-tuning done in order to make the dataset creation process more efficient (Sec. “Fine-tuning for efficient storage”).

### Data collection framework: ExaMon

ExaMon is a holistic framework for HPC facility monitoring and maintenance^12^, designed for very large-scale computing systems, such as supercomputers. At its core, there are software components devoted to gathering data from several sensors distributed among the entire system: these components collect the data and deliver them to upper layers of the infrastructure, using the lightweight MQTT protocol (http://docs.oasis-open.org/mqtt/mqtt/v3). The collector components, also called *plugins* are connected to both hardware (HW) resources and other software (SW) modules, for instance, workload and resource managers–in particular, SLURM^13^ is the resource manager adopted at CINECA–and diagnostic mechanisms. Following the standard MQTT practice, plugins are organized as producers of data, which is then gathered by subscriber entities registered on the agreed communication channels; multiple message brokers are charged with collecting the information coming from the publishers and sending it to the storage areas. Internally, ExaMon is endowed with a NoSQL database (DB), namely Apache Cassandra (https://cassandra.apache.org); for a more efficient handle on time-series data, a companion time-series DB is present (KairosDB, https://github.com/kairosdb/kairosdb).

Among the metrics obtained from HW sensors, there are the CPU load of all the cores in the supercomputing nodes, CPU clock, instructions per second, memory accesses (bytes written and read), fan speed, the temperature of the room hosting the system racks, power consumption (at different levels), etc. Concerning the SW-based plugins, the information provided by the workload manager consists of the job request (e.g., job id, job name, job user, job partition/queue), the requested resources (number of requested nodes, requested cores, requested GPUs, etc.), and the resources actually used. Additionally, diagnostic tools generate alarms and warning messages used by system administrators to check the system state, and ExaMon covers these data sources as well. The same holds for data-center infrastructure sensors, which cover the computer room air conditioning (CRAC) and water chiller system operational parameters and power consumption. The extremely varied nature of the collection is a key strength of ExaMon, as it obtains a very detailed overview of the HPC system and its many components, with a very fine granularity. With this information, it is possible to build a precise digital twin of the supercomputer, which can then be used for various tasks, from modeling and predictive purposes to automation and optimization goals. ExaMon has been deployed on CINECA machines since 2017^11^, albeit over the years it has been subjected to continuous improvements (increased scalability and resilience) and extensions (additions of new plugins).

### Dataset preparation

To produce the dataset, we first extracted all data related to Marconi100 from ExaMon; the data was then processed and transformed into a partitioned Parquet dataset. All the steps can be reproduced by using the shared code, and are described more in-depth in the rest of the section. The extracted data was processed in multiple ways, with three main goals:

1. making access to the data as simple and efficient as possible;
2. preserving original information “as is”, with as little processing as possible;
3. ensuring privacy compliance.

The first goal entails a balance of multiple facets: minimizing storage footprint, with minimal hindrance to retrieval performance; but also organizing data in a clear yet flexible way, enabling modular distribution and use. Parquet (https://parquet.apache.org/) is the technology that was chosen to produce the final dataset, it is popular in the Big Data context and it was capable, with opportune tuning, to satisfy the aforementioned requirements. It allowed compressing the data significantly, without particular deterioration in other regards.

In order to prepare the final datasets (those uploaded in Zenodo) we performed two main actions: i) extraction of the data from the ExaMon monitoring system; ii) processing of the extracted data (removing missing data, make the times uniform, anonymization of sensitive data, etc.), which is described in Section “Data Processing”. The output of the first step (data extraction) is a set of CSV files. These CSV files are then processed (second step) to obtain the final dataset. As the Parquet format is more suited for long-term storage of large amounts of data, we opted to transform the initial CSV file into the Parquet datasets that have been uploaded to Zenodo in the end.

The data was extracted by querying ExaMon separately for each day of each metric, filtering for Marconi100 data only (some metrics are monitored for multiple CINECA clusters). The covered time interval spans from 2020-03-09 to 2022-09-28 (included), for a total of 934 days. The output of this step is a collection of CSV (Comma-Separated Values) files, compressed with GZIP (the notorious single-file/stream lossless data compression utility, http://www.gzip.org/) and organized in a folder structure with this hierarchy: plugin, metric, daily CSV files. This was the starting point for the following steps.

### Data processing

The processing was carried out in two sub-steps. Firstly, we cleaned the data from redundant metrics obtaining an initial Parquet dataset. Redundant information was originally present due to multiple changes in the data collection infrastructure and storage configuration that happened during the course of the long period covered by this data dump. The data that was removed due to redundancy is reported in the description of the plugins (plugin-level metadata, e.g. facility) or in the description of the single metrics (metric-level metadata, e.g. unit measure). In the second and last step, starting from the first Parquet dataset, another one was created, with timestamp truncation for some plugins, anonymization, and minor fixes and refinements.

The vast majority of metrics have timestamps expressed with the precision of one second (i.e., the milliseconds and similar sub-second intervals are not recorded), but some plugins do possess sub-second precision. However, these values are not significant, as they are the consequence of delays in the data collection process and of the mechanisms adopted to mark the timestamp of the data. The removal of this additional but not useful information allows for more homogeneity, which results in both easier access (consistent format for timestamps) and lower storage footprint (mainly due to Parquet encoding leveraging data regularity). Thus, we opted for truncating all timestamps to the second precision, discarding milliseconds and other negligible time differences.

Afterward, sensitive data was identified and treated accordingly. This includes data related to system administrators’ comments (Nagios), data related to users’ jobs (SLURM “job_id” metric and the job table), and the identifiers of the compute nodes (found in many plugins). For the Nagios plugin, most of the metrics were dropped entirely, as i) it was almost impossible to make them completely anonymous and ii) very little value for further analysis remained after the removal of sensitive information. The removed data is a very small fraction of the overall amount of data. The sensitive data in SLURM and the job table is the one related to job identifiers (job_id) and related tags (array_job_id, dependency), user identifiers (user_id), and others (Quality-of-Service and partition). This information is provided by legitimate users of the supercomputer as part of their agreement for receiving access to the machines. This anonymization process has no relevant negative impact, as we still provide documentation about the relative position of the nodes in the room (https://gitlab.com/ecs-lab/exadata/-/blob/main/documentation/racks_spatial_distribution.md). Each of the group of potentially sensitive data (that is, data related to i) system administrators’ comments, ii) users’ jobs, and iii) the identifiers of the compute nodes) was rendered anonymous by transforming the values to corresponding random integers.

### Fine-tuning for efficient storage

**Fig. 1:**
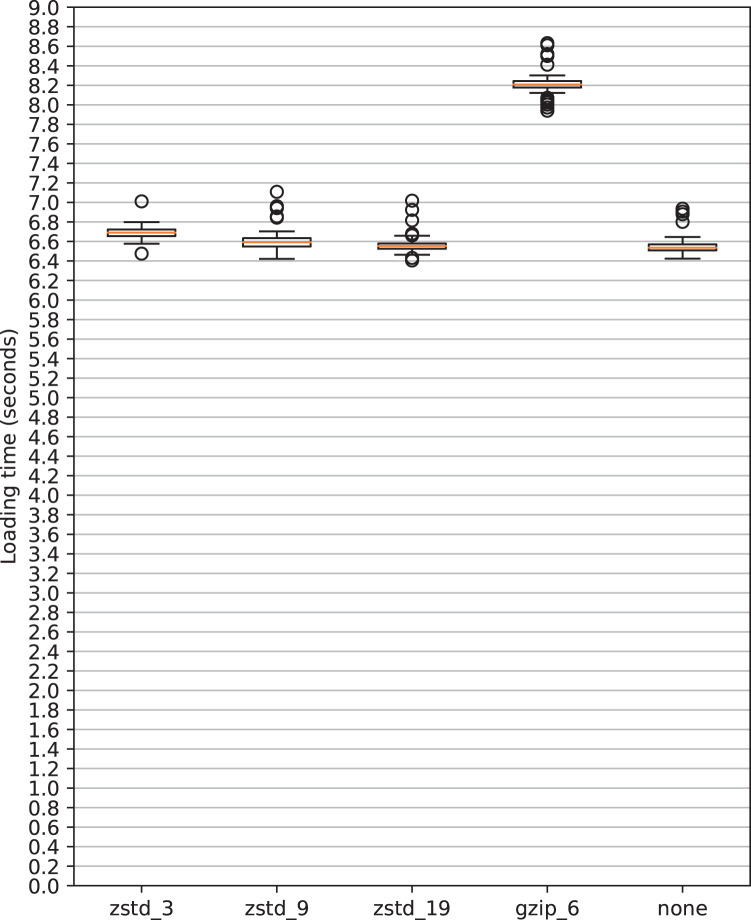
Loading times comparison across compression configurations (Parquet). The data is 4 months of gpu0_core_temp (IPMI), from May to August 2022 (included), retrieving the “timestamp”, “value” and “node” columns. Memory usage by the Pandas Dataframe is 6.55 GB in all cases. The PyArrow io_threads and cpu_count were both set to 8 (Intel Xeon Gold 5220 CPU). The data was loaded using the PyArrow Dataset API.

As aforementioned, we employ Parquet (version 2.6) as the data file format for storing the supercomputer data. Parquet is a flexible column-oriented storage technology, developed for analytics usage. We used PyArrow (https://arrow.apache.org/) (version 9.0.0) and its Dataset API to handle the dataset. The most important parameters in our case are the compression algorithm and compression level. These, along with the encoding schemas used by Parquet and opportune sizing of the row groups, enabled significant storage savings. To manage the compression rate we performed several preliminary experiments with smaller subsets of the data, specifically, we tried different levels of the zstd compression (commonly referred to as Zstandard Compression Format (https://github.com/facebook/zstd/blob/dev/doc/zstd_compression_format.md). After having evaluated the resulting storage size and access times, the chosen compression is zstd level 9. A higher zstd level implies (in general) a reduced storage space, at the cost of slower writing speed; retrieval speed is nearly the same across levels. In Fig. 1 loading times across some compression configurations are compared (including no compression). In the top subfigure (a) the loading times (in seconds) are reported for different compression schemes–gzip clearly offers the worst performance while zstd allows loading times very close to the no-compression applied case. In the bottom part of the figure, we report instead (as a table) the storage space required for the different compression schemes shown above; again, noticing the better performance of zstd is straightforward. Another important implementation decision is the partitioning hierarchy, which was set to (in order): year-month (e.g. 2022-05), plugin, and metric. This setting resulted in chunks small enough to be distributed with Zenodo while avoiding excessive fragmentation. More on this in the following Sections “Data Records” and “Usage Notes”.

Parquet is usually employed with a static schema for the data, expecting it to be the same for all partitions. Here, instead, we created a single partitioned dataset where metrics of different plugins have different schemas. This was overcome by using as a schema the union of all the plugin-specific schemas. An additional challenge is posed by the “value” column having different natural data types for different metrics, as PyArrow and Parquet associate just one specific data type to a given column. This was solved by introducing a small ad-hoc tool, which, on top of PyArrow, retrieves groups of metrics separately, based on the “value” data type, finally merging them by using one common data type for that column. The tool also handles other boilerplate optimization, presenting to the user a straightforward interface to load a subset of the data into a Pandas DataFrame. More about this can be found in the “Usage Notes” section.

## Data Records

The Marconi100 dataset is distributed via Zenodo as 12 different datasets^14–25^, as shown in Table 1; the table reports the official digital identifier (DOI) provided by Zenodo at submission time, the corresponding time period, and the (compressed) dataset size. Later months contain more data due to updates to the monitoring infrastructure happening during the life of Marconi100.

**Table 1:** The dataset is divided among different datasets, all hosted on Zenodo and corresponding to different time frames.

Zenodo dataset (DOI)	Included months	Total size (GB)
Dataset 1^14^	from 20-03 to 20-12 (included)	44.6
Dataset 2^15^	from 21-01 to 21-06 (included)	45.3
Dataset 3^16^	from 21-07 to 21-09 (included)	41.7
Dataset 4^17^	from 21-10 to 21-12 (included)	44.9
Dataset 5^18^	from 22-01 to 22-02 (included)	24
Dataset 6^19^	22-03	31.5
Dataset 7^20^	22-04	33.4
Dataset 8^21^	22-05	33.2
Dataset 9^22^	22-06	27.7
Dataset 10^23^	22-07	31.4
Dataset 11^24^	22-08	37.9
Dataset 12^25^	22-09	34.1
Time-aggregated^26^	from 20-03 to 22-09 (included)	24.8

Here the DOIs, time periods and sizes are reported. The sizes are relative to the whole datasets, download can be done with finer granularity (i.e. months, racks).

It is stored as a partitioned Parquet dataset, with this partitioning hierarchy: *year_month* (“YY-MM”), *plugin*, *metric*. The naming convention is the Hive one, with a folder named “column = value”. The data is distributed as tarball files, each corresponding to one of the 31 months of data (first-level partitioning, *year_month*).

The collected data is generated by a monitoring infrastructure working on unstructured data (to improve efficiency and scalability); however, this data has been organized in a structured manner to simplify its usage by future users. The simplest way to understand how to access the data is to refer to the companion software modules released together with the dataset itself, but we provide a synthetic description in this manuscript as well. There are 573 metrics in total, and each one is a table, with the schema of the records defined by the corresponding plugin. The partitioning columns (*year_month*, *plugin*, *metric*) are present for all plugins; these columns can be used to access specific portions of the dataset, according to the desired plugin, specific metric within the plugin, and time period (selecting year and month).

The exact metrics for each plugin are described in detail in the companion code repository referenced above (https://gitlab.com/ecs-lab/exadata/-/tree/main/documentation), and we have avoided listing them here for clarity’s sake. However, Table 2 reports the various plugins and provides a synthetic description; the first column is the name of the plugin, the second and third ones contain the number of metrics (tables), and the number of plugin-specific columns for the plugin and the final column provides a high-level summary of the information pertaining to the plugin. All but the job table have at least the *timestamp* (that reports the point in time where the data measurement was collected) and *value* (the actual measured value) columns. The job table has a different structure–due to the internal functioning of the plugin collecting job information and how this information is stored on the supercomputer–and aggregated information about the jobs are reported, such as submission, start and completion times, the number of requested resources, resources actually used, submitting users, etc.

**Table 2:** Description of the plugins.

Plugin	#Metrics	#Plugin-specific columns	Description
Vertiv	25	1	Mainly collects data from the air-conditioning units (CDZ) located in room F (Marconi 100) of Cineca. The plugin uses the RESTful API interface available on the individual devices to extract the most interesting metrics.
Schneider	164	1	Dedicated data collector designed to acquire data from an industrial PLC by accessing its HMI module (from Schneider Electric). The PLC controls the valves and pumps of the liquid cooling circuit (RDHx) of Marconi 100. It consists of two (redundant) twin systems controllable by two identical HMI panels, Q101 and Q102.The ExaMon plugin extracts and stores all the metrics available on both panels.
IPMI	104	1	Collects all the sensor data provided by the OOB management interface (BMC) of cluster nodes.
Ganglia	177	1	Connects to the Ganglia server (gmond), collects and translates the data payload (XML) to the ExaMon data model.
Logics	37	2	Data collection system already installed at Cineca. It is specialized for collecting power consumption data from equipment in the different rooms, typically using multimeters that communicate via Modbus protocol. The ExaMon plugin dedicated to collecting this data interfaces to the Logics database (RDBMS) via its REST API. NOTE: Since the translation process is fully automated, the same inconsistencies present in the original db may result in the ExaMon database: e.g., metric names in the Italian language, units of measure as metric name, etc.
Weather	10	0	Collects all the weather data related to the Cineca facility location (Casalecchio di Reno) using an online open weather service (https://openweathermap.org).
Nagios	1	5	Interfaces with a Nagios extension developed by CINECA called “Hnagios”, collects and translates the data payload to the ExaMon data model.
SLURM	54	4	Collects aggregated data from the SLURM server; these information is gathered through ad hoc scripts created by CINECA system administrators.
Job table	1	89	Collects information regarding the jobs executed on the cluster (and store in the SLURM database); the information collected are those provided by users at submission time.

“# Metrics” indicates the number of metrics (tables) for that plugin; “# Plugin-specific columns” is the number of columns for the metrics in that plugin (excluding *timestamp*, *value* and the partitioning columns). SLURM is an acronym referring to the job dispatcher employed at CINECA, that is the Slurm Workload Manager.

**Fig. 2:**
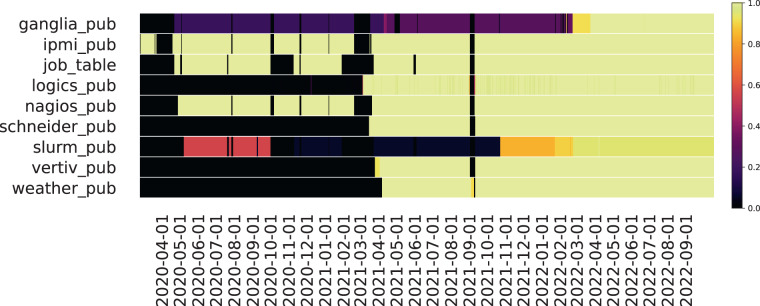
Number of metrics over time (per plugin). Yellow indicates the maximum number of metrics relative to that plugin, and black is the minimum.

**Fig. 3:**
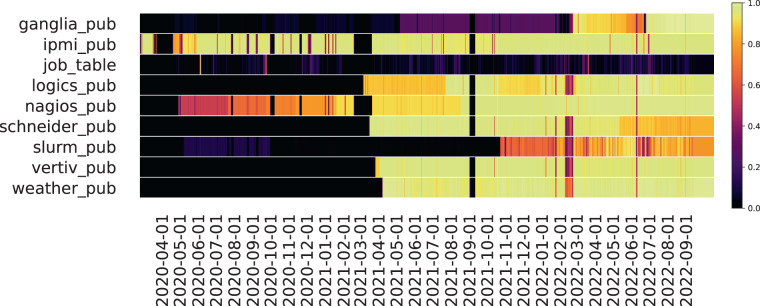
Number of samples collected on a daily basis (per plugin). Yellow indicates the maximum number of samples relative to that plugin, and black is the minimum.

Over the years, many metrics have been added to the monitoring infrastructure (by changing the behavior of ExaMon plugins) and it is, therefore, possible that more recent time periods possess a more numerous set of measurements; a synthetic overview of the number of metrics collected for each plugin over the entire data period can be found in Fig. 2, where different colors indicate the normalized number of metrics per plugin. Additionally, the amount of measurements collected every day has increased over the years, thanks to the addition of new metrics and to the improved reliability of ExaMon, which led to fewer periods with missing data due to monitoring system unavailability. This can be observed clearly in Fig. 3 that reports the cumulative (normalized) number of samples gathered each day for each different plugin; yellow hues indicate a higher number of daily samples, black/darker hues indicate a lower number of samples.

Starting from the main dataset, an additional one^26^ was produced for the “Unsupervised Anomaly Detection” use case, with one Parquet file for each node. The data is distributed in tarballs, each one including all the files relative to the nodes contained in a given rack. For each file, the rows represent periods of 15 minutes, with the columns being aggregations (average, standard deviation, min, max) over all the IPMI metrics that are available for the node; an additional column contains anomaly labels from Nagios. More details about this data can be found in the “Technical Validation - Unsupervised Anomaly Detection” section. The purpose of this separate dataset was to save pre-processing computational time for the future user of the dataset and to demonstrate the potential of statistical representation of the raw data described above.

## Technical Validation

In this section, we describe two use cases that demonstrate how the dataset can be exploited: thermal hazard prediction (Sec. “Technical Validation - Thermal Hazard Detection and Prediction”) and unsupervised anomaly detection (Sec. “Technical Validation - Unsupervised Anomaly Detection”). Admittedly, this pair of examples has only illustrative purposes, as many more types of analysis can be performed using the data. For instance, job power/energy/thermal prediction models can be trained (e.g.^27,28^), novel pricing and accounting schemes can be devised (similarly to^29^), optimal predictive cooling algorithms can be designed (e.g.^30^) as well as fault classification and root cause analysis^31,32^.

We selected the two use cases as they belong to a research area currently under heavy investigation, as predictive maintenance and automated resource management are crucial topics for Exascale supercomputers and scalable datacenters. All the analyses were conducted using the dataset described in this paper and published in the Zenodo repositories, to fully demonstrate how these datasets can be beneficial to other practitioners. The source code used is publicly available: https://gitlab.com/ecs-lab/exadata.

### Thermal hazard detection and prediction

A datacenter consumes a large amount of electrical power (in the range of megawatts), which gets completely transformed into heat^33,34^. Therefore, although a datacenter contains sophisticated cooling systems, minor thermal issues/anomalies can potentially originate thermal hazards. Thermal hazards are detrimental to datacenter operations as they can lead to IT and facility equipment damage as well as an outage of the datacenter, with severe societal and business losses^35,36^.

Therefore, predicting the thermal hazard/anomaly is critical to prevent future disasters. To do this, we used the inlet temperature of the computing nodes’ monitoring data. In the public code repository the simplified version of the datacenter thermal hazard detection and prediction model implemented in Python can be found. The model consists of four main steps: (i) querying the dataset, which provides a targeted subset of the dataset for the study. (ii) pre-processing and dealing with missing data. In this step, we can do any data transformations that are essential for the target study. For example, in this study, first, we pivoted the dataset such that the row indexes are DateTime and columns are inlet temperature of Marconi 100 compute nodes (a conversion that can be envisioned as going from many rows to many columns). Next, based on the studies conducted in^33,34,37,38^, we know 10 minutes sampling rate will preserve room-and-node-level thermal transient but discard computing-component-level ones, so we reduced the sampling rate of the dataset from 20 seconds to 10 minutes with a moving average approach. Then to deal with missing data, we used an interpolation algorithm taken from a standard Python library (Pandas)^39^. (iii) Dataset Annotation, based on the data analysis of the inlet temperature of the computing node in normal and abnormal production conditions of the datacenter in a labe_gene class, we proposed a statistical rule-based method for generating the thermal-hazard label. (iv) Machine Learning (ML) step, composed of (a) Dataset Creation, obtained by splitting the dataset into a training set and test set, with a split ratio of test-size = 0.2 and train-size = 0.8. (b) Data Standardization, transforming data into a standard format, helps to improve training performance. (c) Machine Learning Model, we used support vector classification, a supervised learning model. In the ML step, we used sklearn libraries^40^. The performance of the proposed thermal hazard prediction model reached an F1-score (weighted average) of 0.94.

### Unsupervised anomaly detection

Anomaly detection is essential in high-performance computing (HPC) systems as it helps to identify unusual or unexpected behavior in system and application performance, which can indicate problems or potential issues^41^. An example of an anomaly in an HPC system is a sudden increase in resource usage, a drop in performance, or an unexpected error. Anomaly detection can help identify these issues early on to be addressed before they cause serious problems or disruptions. This can help to ensure the smooth operation of HPC systems and applications and prevent costly downtime or lost productivity. Anomaly detection is crucial for HPC environments because these systems often have large amounts of data and complex relationships, making it difficult to identify problems manually. Anomaly detection algorithms can analyze this data and identify patterns or deviations that may indicate an issue. These algorithms can also be configured to alert administrators or take other automated actions in response to detected anomalies, helping to ensure that issues are addressed quickly and effectively.

The dataset presented in this paper can be used to train an anomaly detection algorithm. For anomaly detection, data is first aggregated into 15-minute time intervals (for each interval, we have min, max, average value, and standard deviation). The data is aggregated in this manner because we exploit the alarms employed by system administrators to identify malfunctioning situations; these alarms are gathered by the Nagios plugin with a 15-minutes frequency, thus the aggregation period for the anomaly detection task. Using a higher sampling rate (as allowed by the other data sources involved) would not let us actually validate any anomaly detection approach, as we are restricted by the production of the anomalies via Nagios. The data is structured as a time series, with rows representing a single time stamp and columns representing the features in the dataset. The aggregation and pivoting code that transform the raw dataset into the dataset suitable for training anomaly detection models are available in the code repository.

In the code examples, we have adopted an unsupervised anomaly detection algorithm RUAD^42^ and trained it on a single compute node. We have used 80 percent of all data as a train set and (chronologically) the last 20 percent as a test set. Then we evaluated the area under the receiver operator characteristic curve as the measure of performance on the test set. The real labels used in the evaluation of the anomaly detection approach come from the Nagios software module used by system administrators to check the status of various services in the supercomputer, and to flag nodes that are in an anomalous state and thus need further inspection (nodes flagged as anomalous are put in an “offline” state until fixed or examined, and cannot be allocated to users’ jobs until returning in the normal state). Nagios is one of the sources of information collected by ExaMon, hence we use it to (implicitly) annotate the dataset. The labels used in the testing phases of the anomaly detection approach are thus those obtained via Nagios. On the selected node, the classifier achieves the area under the curve (AUC) of 0.57. The code base contains no plots or any kind of visualization, as it only includes the necessary libraries to execute the analysis.

## Usage Notes

The datasets have been devised to offer the possibility to do analysis and experiments to the wider HPC research community. To this end, we aim at offering a suite of companion software tools to facilitate access to the data–in addition to the raw data, which can nevertheless be directly accessed using any technology that can handle Parquet (e.g. Spark, Dask). The companion software is available at the following web repository https://gitlab.com/ecs-lab/exadata/-/tree/main/parquet_dataset/query_tool and it is distributed as open-source code (see the code for details). This tool offers a simple way to select a subset of the data in the form of a Pandas DataFrame while handling boilerplate optimizations and some specific design choices (e.g., the “value” column having multiple possible data types). The distribution of the data was designed in order to allow selective download of reasonably sized (a few GB) and self-contained fragments (i.e. months of data). Multiple fragments can be placed in a common folder, in order to obtain a custom-sized Parquet dataset. Examples of how to use the tool can be found in the code repository. In particular, the code corresponding to the use cases described in Section “Technical Validation” can be found there as well. Thermal hazard detection and prediction (https://gitlab.com/ecs-lab/exadata/-/tree/main/examples/thermal_hazard_detection_and_prediction); anomaly detection (https://gitlab.com/ecs-lab/exadata/-/tree/main/examples/anomaly_detection).

## Data Availability

The code we have used to generate this dataset has multiple sources, albeit all being publicly available. As defined previously, the data is collected through a monitoring infrastructure called ExaMon, which we have developed and deployed on the CINECA infrastructure. The code is publicly available and can be used in different supercomputing facilities (https://github.com/EEESlab/examon). The libraries and software modules used to process the data streams and obtain the dataset presented here are the following: • examon-client • NumPy (1.23.1) • Pandas (1.5.1) • PyArrow (9.0.0) In addition, in the source code repository (https://gitlab.com/ecs-lab/exadata) it is possible to recover the list of software modules used to access the data and to perform the analysis described in Sec. “Technical Validation”.
